# Super-Resolution Generative Adversarial Network Based on the Dual Dimension Attention Mechanism for Biometric Image Super-Resolution

**DOI:** 10.3390/s21237817

**Published:** 2021-11-24

**Authors:** Chi-En Huang, Yung-Hui Li, Muhammad Saqlain Aslam, Ching-Chun Chang

**Affiliations:** 1 AI Research Center, Hon Hai Research Institute, Taipei 114699, Taiwan; a3285556aa@gmail.com; 2 Department of Computer Science and Information Engineering, National Central University, Taoyuan 320317, Taiwan; saqlain@g.ncu.edu.tw; 3 Department of Computer Science, University of Warwick, Coventry CV4 7AL, UK; ching-chun.chang@warwickgrad.net

**Keywords:** super-resolution, attention mechanism, generative adversarial network, biometric recognition

## Abstract

There exist many types of intelligent security sensors in the environment of the Internet of Things (IoT) and cloud computing. Among them, the sensor for biometrics is one of the most important types. Biometric sensors capture the physiological or behavioral features of a person, which can be further processed with cloud computing to verify or identify the user. However, a low-resolution (LR) biometrics image causes the loss of feature details and reduces the recognition rate hugely. Moreover, the lack of resolution negatively affects the performance of image-based biometric technology. From a practical perspective, most of the IoT devices suffer from hardware constraints and the low-cost equipment may not be able to meet various requirements, particularly for image resolution, because it asks for additional storage to store high-resolution (HR) images, and a high bandwidth to transmit the HR image. Therefore, how to achieve high accuracy for the biometric system without using expensive and high-cost image sensors is an interesting and valuable issue in the field of intelligent security sensors. In this paper, we proposed DDA-SRGAN, which is a generative adversarial network (GAN)-based super-resolution (SR) framework using the dual-dimension attention mechanism. The proposed model can be trained to discover the regions of interest (ROI) automatically in the LR images without any given prior knowledge. The experiments were performed on the CASIA-Thousand-v4 and the CelebA datasets. The experimental results show that the proposed method is able to learn the details of features in crucial regions and achieve better performance in most cases.

## 1. Introduction

The number of IoT devices worldwide is estimated to almost triple from 8.74 billion in 2020 to more than 25.4 billion IoT devices in 2030 [1]. It can be foreseen that a large amount of data will be collected and the computational power required by cloud computing will rise accordingly. Considering the applications envisioned for IoT (smart cities, homes, retail, etc.), the security issues must be handled with great care. Since the sensors will be the primary device to accept the data from the environment in the cloud computing framework, they may become the first targets to be attacked. As the technology advances, the traditional password authentication is expected to be gradually replaced by the biometric recognition system. Recently, various biometric systems have been built on IoT devices [2,3,4,5], which may enhance the security of the loT network as well as decrease the probability of the sensor node being compromised, and present the advantage of biometric-based IoT (BiometricIoT). Moreover, existing research shows that applying multiple biometrics for authentication helps increase recognition accuracy, making biometric approaches a more reliable choice for securing the IoT.

However, one factor that is crucial to the biometric accuracy is the resolution of the input image. It heavily depends on several factors of the optical factors of the sensor, such as the focal length, field of view, depth of focus, and the combination of them. Due to the hardware constraints of IoT devices, the acquired LR image by low-cost IoT sensors will highly downgrade the recognition accuracy and negatively affect the reliability of authentication. So, the aforementioned BiometricIoT technique may encounter physical limitations in practical situations.

In the field of computer vision, the aforementioned issues of reproducing accurate HR images from the corresponding LR counterpart are referred to as SR [6]. SR is one of the most popular research areas and has received extensive consideration in the computer vision research community, which also has a broad range of applications for different biometric modalities. The SR method is generally classified into two categories based on the number of input images: single-image resolution (SISR) and multi-image super-resolution (MISR) [7]. According to the SISR framework, we can super-resolve the corresponding HR image from the given LR input; hence, it is able to mitigate the problem caused by the hardware constraint of BiometricIoT and further enhance the accuracy of biometric authentication and identification in the cloud computing environment.

In recent years, methods based on deep learning have made significant progress over the traditional SR methods. In particular, the SR technique based on GAN [8] (SR-GANs), which is one of the most famous frameworks of the deep learning method, has demonstrated outstanding performance for the tasks of computer vision. The SR-GAN enhances the quality of LR images via the various loss functions, which have been proposed to improve the image quality from various perspectives. Currently, several existing SR works proposed the attention mechanism in order to enhance the performance [9,10]. In particular, a few works have proposed the mask attention to generate the accurate SR image for the downstream task [11,12,13], such as medical segmentation or biometric recognition. However, most of the work still request the additional information to manually label the ROI region to denote the ground truth attention region. Such an attention technique may need human intervention to give the ground truth for every image, which increases the cost of dataset pre-processing. It also slows down the inference process because the attention regions need to be estimated before the model starts the SR process.

In this work, we proposed the dual-dimension attention approach to enable the model to learn the corresponding ROI automatically by inspecting the interdependence relationship between different channels of feature maps and force GAN to focus on the appearance difference of ROI between the super-resolved image and the corresponding HR image. According to the feedback of discriminator, the generator will be trained to discover ROI by itself and learns the structure and texture correspondence between LR and HR images. We named the proposed model dual-dimension attention SRGAN(DDA-SRGAN), which is a GAN-based framework employing the attention mechanism.

In summary, the main contribution of this paper is as follows:

1. We propose DDA-SRGAN, a GAN-based SR framework using a dual-dimension attention mechanism for image SR. It will automatically locate the ROI region without given prior knowledge and further enhance the feature details inside the corresponding region.
2. Our proposed attention module will capture the ROI features in the LR image by considering the interdependence of feature maps with respect to the spatial dimension and the channel dimensions. Furthermore, the DDA mechanism forces the generator in GAN to generate more informative SR images and enhance the performance of the downstream tasks.
3. We have built an SR framework for enhancing the existing authentication system of BiometricIoT, and further decrease the possibility of compromising sensor.

We obtained a series of comparable results with a higher verification rate (VR) in most cases. On the dataset Celeb-Attribute, it is able to achieve 84.24% VR with a 5% false-accept rate (FAR), and the equal error rate (EER) is 6.84%. On the dataset CASIA-Thousand-v4, it is able to achieve 92.7% VR with 1% FAR and attains comparable performance with 2.24% EER. Such an error rate is lower than the state-of-art (SOTA) in the GAN-based SR model.

## 2. Literature Review

Among the recently proposed SR approaches, most of the GAN-based methods generate SR images with high quality. Moreover, we found that the attention mechanism is also play a big role in such methods. So, we mainly concentrate on the previous work associated with the GAN-based approaches as well as the attention mechanism in this section.

The seminal work based on the deep-learning carried out by SRCNN [14], which determine the mapping from LR to HR image in an end-to-end (E2E) manner and obtained outstanding performance that is superior to the traditional works. After that, various network architectures have been proposed, such as residual blocks [15], residual-dense networks [16], Laplacian pyramid structure [17], densely connected network [18], recursive learning [19,20], and deep back-projection [21]. Specifically, Kim et al., proposed VDSR [15], which introduced the residual learning to stabilize the training procedure of the deeper model. With the competitive performance, DRCN [19] utilized deep recursive network to saving the memory usage by consolidating intermediate outcomes, and DRRN [20] was makes use of the residual learning to further stabilize the training procedure. EDSR and MDSR (its multiple-scale factor versions) [22] is the latest technology based on PSNR metric. Tong et al., introduced the SRDenseNet [23], which uses the residual-dense networks with the skip connection to enhance the combination of features on different levels. Based on the backbone of DenseNet [18], Yulun Zhang et al., further proposed RDN [16], which merges densely connected convolutional networks with residual connections, and then combine the hierarchical features from different convolutional layers to present the generated image.

In order to pay more attention to the visual quality of the generated images, a perception-driven method is proposed to improve the visual quality of SR results. As a pioneer of the GAN-based framework to solve SR, Christian Ledig et al. [24] proposed SRGAN employing perceptual loss [25,26] along with an adversarial loss to generate the photorealistic images. In the GAN-based framework, the perceptual loss makes the generated image more proper for the human visual system. Despite having a low score on standard quantitative measures such as PSNR and SSIM, these images are more visually convincing. EnhanceNet [27] is also based on GAN but uses a different architecture. As the name suggests, ESRGAN enhances SRGAN. It proposes a new block with a larger capacity called RRDB. In addition to removing the BN layer, residual scaling and smaller initialization are also employed to facilitate the training of very deep networks. The discriminator uses relativistic average GAN, which determines “whether one image is more accurate than the other” instead of whether one image is original or fake. Moreover, in the perceptual loss, the VGG [28] characteristics are practiced before activation rather than later as in SRGAN.

Even though SRGAN and Enhancenet can reduce blur and excessive smoothing artifacts to some extent, their predicted outcomes may not be conscientiously reconstructed and may generate unpleasant artifacts. By removing undesirable modules from conventional residues networks, Lin et al. [22] suggested EDSR and MDSR, and they have made notable progress. However, most of these methods have a limited network depth, which has proven to be very important in visual recognition tasks [29] and can reach about 1000 layers. Only stacking residual blocks in MDSR [22], deep networks can barely achieve improvements. Furthermore, most of these approaches employ the channel-wise features uniformly, preventing the better discriminative capacity for different traits. Additionally, Nathanael Carraz Rakotonirina et al. [30] proposed ESRGAN+, an enhancement of ESRGAN by putting RRDB into RRDRB through further adding cascading paths in internal dense blocks, which will improve the network capability. The technique of providing finer details at a high level requires adding Gaussian noise to the main path of the RRDRB structure.

Recently, tentative investigation has been focused on deep neural networks [31,32,33], ranging from image localization and perception in images [34,35] to sequence-based networks [36,37]. It is customarily used in conjunction with a gating function (such as sigmoid) to rescale the feature map. Wang et al. [32] introduced a residual attention network for image classification through trunk-and-mask attention mechanisms. Hu et al. [31] introduced a squeeze and excitation (SE) block to model the channel-wise relationship to gain significant image classification performance advancement. Nevertheless, few researchers have proposed to study the impact of attention on low-level visual tasks (such as image SR). Zhang et al. [9] proposed the very deep residual channel attention networks (RCANs), which use the residual-in-residual (RIR) backbone and channel attention mechanism to adaptively rescale channel-wise features by considering interdependencies between channels. However, the channel attention does not fully investigate the contextual information in each feature map, so the details of the spatial feature may not be able to be recovered. Sanghyun Woo et al. [38] further proposed the convolutional block attention module (CBAM) framework, which includes two attention modules in the residual block to utilize significant features from the spatial and channel dimensions, in order to solve the mentioned issue. However, the inner structure of the attention module is relatively primitive, and we further improve the network structure in our work.

On the other hand, there exists some work using the meta information to control the attention region to further enhance the downstream task performance. Kim et al. [11] proposed a novel facial attention loss, focusing on recovering facial features with more detail, and also introduced a FAN network to extract heat map values to manifest the ROI of facial landmarks. Qingyun Li et al. [12] proposed a novel framework called tumor GAN, which generates the tumor mask by merging the lesion area and the contour area, and then introduced a regional perceptual loss to enhance the performance of the discriminator by the given tumor mask. Recently, Huang et al. [13] proposed MA-SRGAN to enhance the ROI feature for the biometric recognition. In such work, the relationship between the SR framework and downstream task has been inspected systematically, and the corresponding mask has been developed according to the domain knowledge of the downstream task. However, the human intervention for labeling the meta information is still inevitable during the training phase.

## 3. Materials and Methods

### 3.1. Proposed Network Architecture for SR

**Figure 1:**
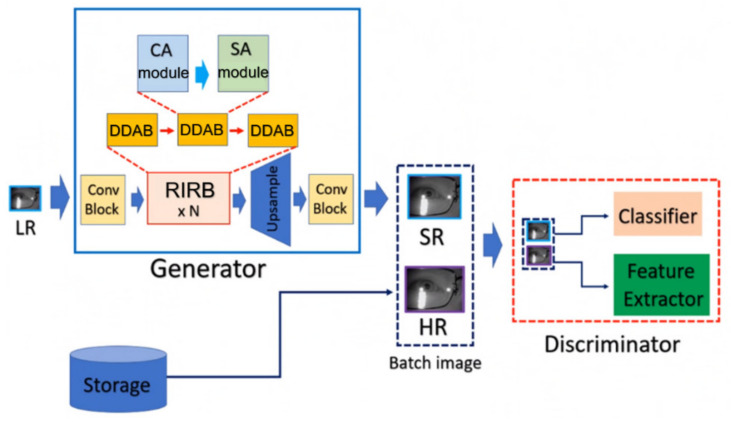
DDA-SRGAN architecture. DDA-SRGAN is a GAN-based approach, which is composed of the generator and the discriminator. In the generator, the ROI feature will be extracted from the LR images by the high-capacity block RIRB, and the informative embedding will be used to generate the accurate SR images by the bicubic-convolution upsampling [14]. On the other hand, the framework is similar to the previous work [30].

In this study, we propose a dual-dimension attention super-resolution (DDA-SRGAN) model. The proposed kernel module in the overall network is dual-dimension attention block (DDAB), which is able to automatically learn to locate the ROI region by inspecting the interdependent relationship between feature maps in the channel dimension as well as the spatial dimension and then extract the discriminative feature inside the ROI for reconstructing the detail information of SR image. Moreover, the DDAB can be the fundamental component of any network backbone for extracting the ROI feature, and the proposed module does not rely on any prior domain knowledge of the downstream task to indicate the ROI region. The flowchart of the proposed method is shown in Figure 1.

#### 3.1.1. Overall Network Architecture

The proposed DDA-SRGAN makes use of the nESRGAN+ [31] as the network backbone due to its high capacity of network, which allows the network to learn stable feature representation by fusing the extracted feature on different levels. In this way, the multi-level network generator can fit the complex pixel distribution and produce high-quality SR images. The generator of DDA-SRGAN mainly consists of residual-in-residual blocks (RIRB), and each RIRB is further composed of dual-dimension attention blocks (DDAB). Suppose the low-resolution image is $Im_{LR}\in {\mathbb{R}}^{H\times W\times C}$ and the corresponding height, weight, and channel denote H, W, and C, respectively. The formal definition of the overall network architecture is given by Equation (1):

(1) $$sr_{embed}=fea_{n}+fea_{0}=\beta \;⊙\;RIRB_{n}\left(\;fea_{n-1}\right)+fea_{0},\;\;where\;fea_{i}=RIRB_{i}\left(\;fea_{i-1}\right),\;i=1,\ldots ,\;n,\;\;fea_{0}=Im_{LR}\;\;$$

where ⊙ is the matrix scalar multiplication operator which takes each element in the matrix (such as feature map) multiplied by the single scalar value; β is the residual scalar to stabilize the network training; $fea_{i}$ is the intermediate feature map of the *i*-th layer output and $fea_{0}$ is the low-resolution image as the input of the block RIRB, and $sr_{embed}$ is the input of the up-sampling layer, which is the last layer in the generator.

**Figure 2:**
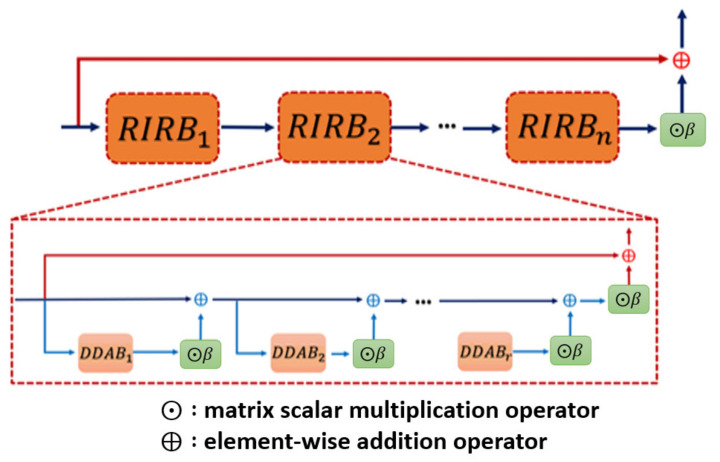
Residual in residual block (*RIRB*) inner structure. Each of the RIRB is composed of several DDAB, which is our proposed kernel module to fulfill the dual dimensional attention mechanism, with the residual scaling for stabilizing the training procedure.

Inspecting the detail of RIRB, the formal definition of the i-th *RIRB* is given by Equation (2):

(2) $$RIRB_{i}\left(fea_{i-1}\right)=\beta \;⊙\;DDAB_{\;i,\;r}\left(\;fea_{i-1,\;r-1}\right)+fea_{i-1,0},\;\;where\;\;fea_{i,\;j}=\beta \;⊙\;DDAB_{\;i,\;j}\left(\;fea_{i-1,\;j-1}\right)+fea_{i-1,\;j-1},\;j=1,\ldots ,\;r\;$$

where $fea_{0,\;0}=fea_{0}$ is the initial input of the block DDAB, and here, the Gaussian noise is omitted in each layer of the DDAB. Moreover, the inner structure as well as the formal description of the *DDAB* will be revealed in the next section. The network architecture of the RIRB is shown in Figure 2.

#### 3.1.2. Attention Mechanism Module

**Figure 3:**
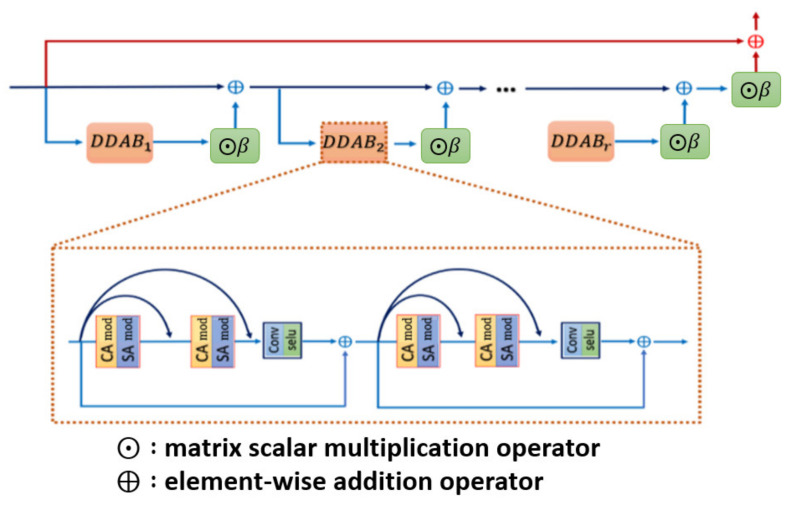
Dual-dimension attention block architecture. Each of the DDAB is composed of the proposed CAM followed by the SAM, which are able to figure out the ROI feature by investigating the cross-channel correlation as well as the inter-channel correlation.

After illustrating the whole picture of network structure, we further inspect the DDAB structure in detail, which offers the attention mechanism to extract the ROI feature with respect to the channel dimension and the spatial dimension, and the inner structure of DDAB is illustrated in Figure 3. Specifically, the DDAB is composed of the channel attention module (CAM) as well as the special attention module (SAM) in the sequential order. So, the feature maps will be processed by the CAM, and the weighted channel scalars will be produced to indicate which feature map is more important than the others. Later, the weighted feature maps will be further processed by the SAM. The contextual feature will be extracted from the weighted feature map, and it produces the weighted feature map in the next layer to further enhance the feature inside the ROI region.

### 3.2. The Kernel Modules of Dual Dimension Attention Block 

#### 3.2.1. Channel Attention Module (CAM)

**Figure 4:**
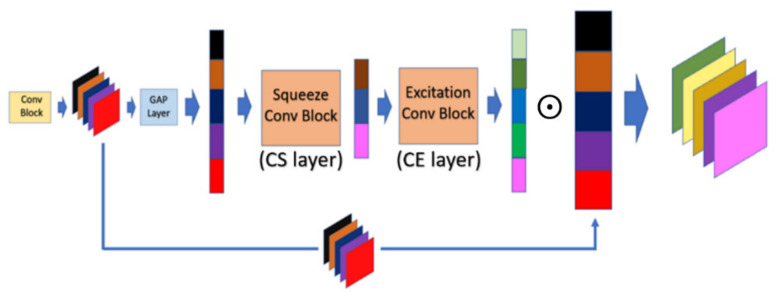
Operation mechanism architecture of channel attention module (CAM).

In CAM, the interrelationship in terms of the channel dimension between the feature maps will be inspected by performing the sequential operations, which are global average pooling (GAP), channel squeeze (CS), and channel excitation (CE). At first, each input feature map will be compressed into the single dense scalar by calculating the average value of the corresponding feature map in the GAP layer. To achieve the robust representation of feature maps, the vector containing all average value will be mapped into the dense vector space by performing the non-linear transformation in the CS layer, and the dense vector will be further mapped back into the higher dimension in the CE layer. The resulting output of the CE layer is called the weighting vector, and it is further normalized by the sigmoid activation function to constrain its range within [0, 1]. Finally, each feature map multiplied by the corresponding scaling factor will present how important the corresponding feature map is between the feature maps. The scaling factor will be automatically calibrated in the training phase without the domain knowledge of ROI. Suppose the height, width, and channel of the input feature map are fh, fw, t, respectively. The formal definition of the *GAP*, *CS*, and *CE* layer can be described by Equations (3)–(5), respectively. Finally, the formal definition of integral *CAM* module is given by Equation (6):

(3) $$GAP\left(fea\right)=\frac{1}{fh\times fw}\overset{fh\times fw}{\underset{i=0}{\sum }}fea_{i}^{u}=\tilde{fea},\;where\;fea\in {\mathbb{R}}^{fh,fw,t},\;u=1\sim t,\;\tilde{fea}\in {\mathbb{R}}^{1,1,t}$$

(4) $$CS\left(fea\right)=\left(W_{cs}*fea\right)+b_{cs},where,\;W_{cs}\in {\mathbb{R}}^{3,3,s},b_{cs}\in {\mathbb{R}}^{fh,fw,s}$$

(5) $$CE\left(fea\right)=\left(W_{ce}*fea\right)+b_{ce},where,\;W_{ce}\in {\mathbb{R}}^{3,3,t},b_{ce}\in {\mathbb{R}}^{fh,fw,t}$$

(6) $$CAM\left(fea\right)=CE\left(CS\left(GAP\left(fea\right)\right)\right)⊙fea$$

where ∗ denotes the convolution operator; W is the convolution kernel; b is the bias value; ⊙ is the matrix scalar multiplication operator; and s≪t in the number of feature maps. Finally, the overall structure of *CAM* is shown in Figure 4.

#### 3.2.2. Spatial Attention Module (SAM)

**Figure 5:**
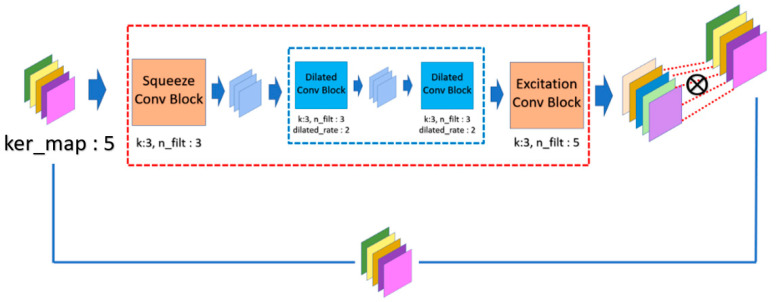
Operation mechanism architecture of spatial attention module (SAM).

In SAM, the inner relationship in terms of the spatial dimension for each feature map will be inspected by performing the sequential operations, which are dilated convolution (DC), CS, and CE. The spatial dimension of the feature map reveals the relationship with the nearby feature extracted by the previous convolution. However, the constrained receptive field limits the capability of feature extraction. So, we make use of the DC layer to further extend the receptive field by increasing the stride, and the contextual information will be extracted with mitigation of the effect of the redundant information. Moreover, we perform a smaller stride of the DC layer to avoid losing the information detail and performing K times DC operation to confirm that the contextual information will finally be extracted. However, it may require very heavy computational resources due to the numerous feature maps. In order to lessen the computational requirement, we compress the information of feature map by wrapping the DC layer between the CS and the CE layer as Squeeze-Net. The resulting output of the CE layer is called the weighting mask, and we further constrain the range of weighting masks to obtain the normalized mask components using the sigmoid activation function. Finally, each feature map will be enhanced by performing the element-wise multiplication with the corresponding mask. The mask component will be automatically calibrated during the training phase without the domain knowledge of the ROI. The formal definition of the *CS*, *CE*, and *DC* layer can be described by Equations (7)–(9), respectively. Finally, the formal definition of a complete *SAM* module is given by Equation (10).

(7) $$CS\left(fea\right)=\left(\;W_{cs}{*}_{1}fea\right)+b_{cs},\;where\;W_{cs}\in {\mathbb{R}}^{3,\;3,s},\;b_{cs}\in {\mathbb{R}}^{\;fh,fw,\;s}$$

(8) $$CE\left(fea\right)=\left(\;W_{ce}{*}_{1}fea\right)+b_{ce},\;where\;W_{ce}\in {\mathbb{R}}^{3,\;3,\;t},\;b_{ce}\in {\mathbb{R}}^{fh,fw,\;t}$$

(9) $$DC\left(fea\right)=\left(\;W_{cs}{*}_{2}fea\right),\;where\;W_{cs}\in {\mathbb{R}}^{3,\;3,\;s},\;dr=2$$

(10) $$SA\left(fea\right)=CE\left(DC_{k}\left(CS\left(fea\right)\right)\right)⊗fea,\;where\;k=1,\ldots ,K\;$$

where ${*}_{dr}$ is the convolution operator with the dilated rate dr; W is the convolution kernel; b is the bias value; ⊗ denotes the element-wise multiplication operator; and s≪t in the number of feature maps. Finally, the overall *SAM* is demonstrated in Figure 5.

### 3.3. Overall Network Loss Function

Since nESRGAN+ [30] is adopted as the network backbone of DDA-SRGAN, all of the loss function used in nESRGAN+ will remain in this work. Such loss functions are also commonly used for enhancing the image quality from different perspectives in the field of SR, and the attention modules of DDAB are optimized by those loss terms automatically.

The overall loss of the DDA-SRGAN network is a combination of perceptual loss, relativistic loss, and L1 pixel loss. Finally, the overall loss value is available by the linear combination of each loss values of the network, and the formal definition is described by Equation (11).

(11) $$L_{G}=\lambda L_{percep}^{befVGG}+\eta L_{adv}^{Ra}+\gamma L_{pixel}^{L1}$$

where λ, η, γ are the weight of individual loss value, and the details of each loss function are given by the following Equations (12)–(16).

(12) $$L_{percep}^{befVGG}=-E_{\begin{array}{c} hr\sim P_{HR}{}_{img}\\ lr\sim P_{LR}{}_{img} \end{array}}∥\varphi_{i,\;j}\left(hr\right)-\varphi_{i,\;j}\left(G\left(lr\right)\right)\;{∥}_{2}$$

where $\varphi_{i,\;j}$ denotes the *VGG*-19 pre-training network, which extracts the feature from *i*-th layer before the *j*-th activation, and the parameters used in this study are i=5,j=4. Finally, the L2 distance between the real image and the generated images is obtained.

(13) $$L_{adv}^{Ra}=-E_{hr\sim P_{HR_{img}}}\left[log\left(1-D_{Ra}\left(hr,sr\right)\right)\right]-E_{sr\sim G\left(lr\right)}\left[log\left(D_{Ra}\left(sr,hr\right)\right)\right]$$

(14) $$D_{Ra}\left(hr,sr\right)=\sigma \left(C\left(hr\right)\right)-E\left[\sigma \left(C\left(sr\right)\right)\right]$$

(15) $$D_{Ra}\left(sr,hr\right)=\sigma \left(C\left(sr\right)\right)-E\left[\sigma \left(C\left(hr\right)\right)\right]$$

where $E\left[.\right]$ is the expected value and $C\left(.\right)$ is the unbounded output of the discriminator, whose range of output value may beyond [0, 1]. Again, we use $\sigma \left(.\right)$, which is the sigmoid activation function, to constrain the value in the range of [0, 1]. It can be seen that $D_{Ra}\left(hr,sr\right)$ determines whether the real image is more real than the average generated images. In contrast, $D_{Ra}\left(sr,hr\right)$ determines whether the generated image is faker than the average of real images. In this way, the discrimination capability will be improved, and the quality of generated image will be further enhanced indirectly.

(16) $$L_{pixel}^{L1}=-E_{lr}∥G\left(lr\right)-hr{∥}_{1}$$

where G is the generator and the $L_{1}$ distance of each pixel value between the SR image and the HR image is obtained.

## 4. Experiments and Results

The proposed DDA-SRGAN can be used in the various fields of computer vision tasks, for example, biometric authentication or medical image enhancement. In this section, in order to evaluate the effectiveness of the proposed model in practical situations, we follow the experimental framework proposed in [13], which means we will conduct experiments of biometric recognition (face and iris) based on SR images, which are rendered by various models, and the biometric recognition accuracy will be used as a practical indicator for SR performance comparison.

In this work, we use the dataset CASIA-Iris-Thousand v4 [39] for iris recognition and the dataset CelebFaces Attributes [40] for face recognition. The specifications of the dataset are described as follows, and detailed information of both datasets is presented in Table 1.

### 4.1. Iris Dataset Specification

CASIA-Iris is the worldwide largest dataset of iris released by the Chinese Academy of Sciences. It contains a total of 20,000 images and 2000 classes, and each class contains 10 HR (640 × 480) eye images. We manually labeled the mask for each iris image in the dataset.

### 4.2. Face Dataset Specification

CelebA is a large-scale human face attributes dataset released by the Chinese University of Hong Kong. The CelebA dataset contains 202,599 images with 10,177 classes, and each class contains various numbers of images. As the number of images contained in each class is unbalanced, we preprocess the dataset so that each class contains 20 images with a resolution of 160 × 200; the resulting number of classes used for experiments is 6000. Note that the dataset contains some factors that make the dataset harder to recognize, such as ill-posed faces or faces with age variation. Due to the enormous diversity of the dataset, the difficulty level of biometric recognition using CelebA is closer to the real-world application.

### 4.3. Partition of Experimental Dataset

In this experiment, the CASIA-Iris-Thousand-v4 [39] and Large-scale CelebFaces Attributes [40] datasets are further divided into the training subset and the evaluation subset, respectively. In the training phase, half of the classes will be used to train the model. In the evaluation phase, we attempt to simulate the practical situation of biometric recognition, which enrolls the high-quality images during the enrollment process in most cases. So, the gallery set contains the HR image as the ground truth, while the probe set contains either the LR image or the SR images generated from the LR images. To this goal, the evaluation dataset was further divided into two subsets: gallery set and probe set. Note that we simulate the resolution downgrade by using the bicubic kernel during the down-sampling procedure, which is the classical method used in most of SR work. So, we simulate each LR image by downsampling the HR image to a quarter of its original image size, and then feed each LR image into various SR models in the training phase as well as the evaluation phase. Finally, detailed information of the iris dataset partition and the face dataset partition are presented in Table 2 and Table 3, respectively.

#### 4.3.1. Iris Dataset Partition

In the training phase, the model is trained with all of the left iris images from all categories, and the training data are also augmented by the horizontal flip, so that the model is able to learn the general feature to generate the right iris images. In the evaluation phase, all of the right iris images are used to evaluate the model performance. In the gallery subset, the first half of images from each category are chosen and the number of the subset is 5000 images. In the probe subset, the remaining images from each category are chosen and the number of images in the subset is also 5000.

#### 4.3.2. Face Dataset Partition

In the training phase, the model is trained with 4200 categories of face images, and the training data are also augmented by the horizontal flip, so that the model is able to learn the general feature. In the evaluation phase, the remaining 1800 categories of face images are used to evaluate the model performance. In the gallery subset, the first half of images from each category are chosen and the number images in the subset is 18,000. In the probe subset, the remaining images from each category are chosen and the number of images in the subset is also 18,000.

### 4.4. Domain Knowledge of Biometrics

#### 4.4.1. Common Procedure of Biometrics

**Figure 6:**
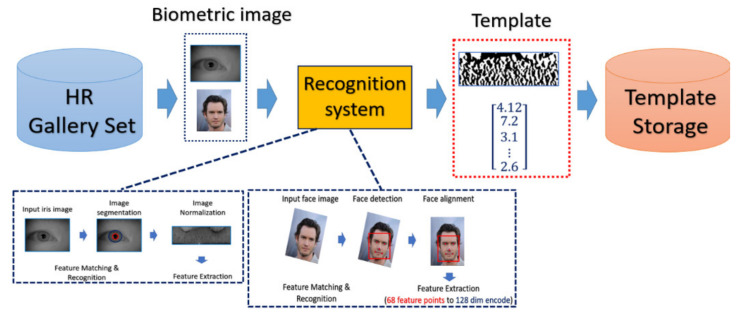
The flow chart of biometric template enrollment stage.

**Figure 7:**
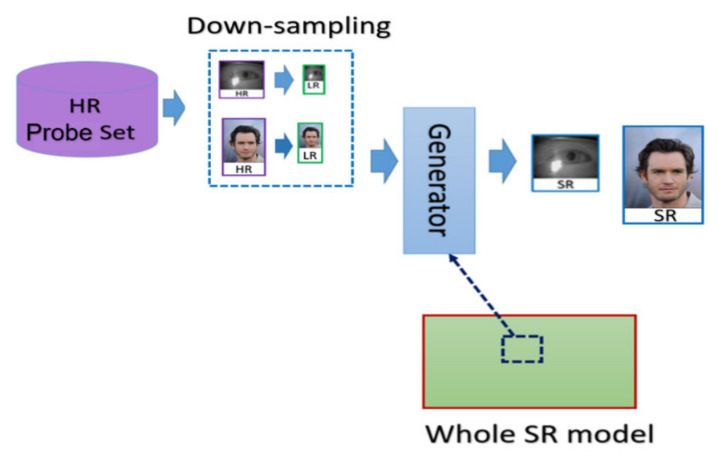
The flow chart of the SR images generation stage.

**Figure 8:**
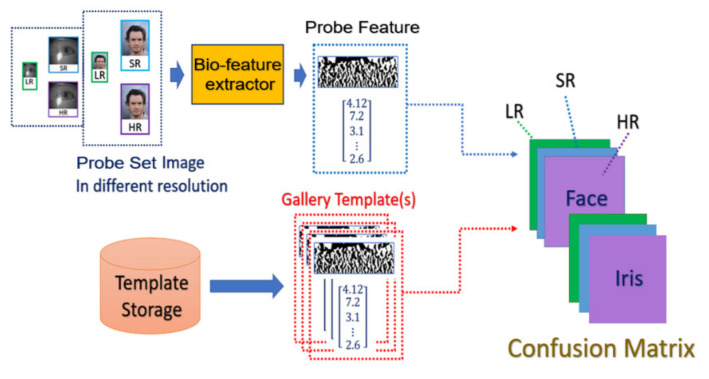
The flowchart of the matching stage.

**Figure 9:**
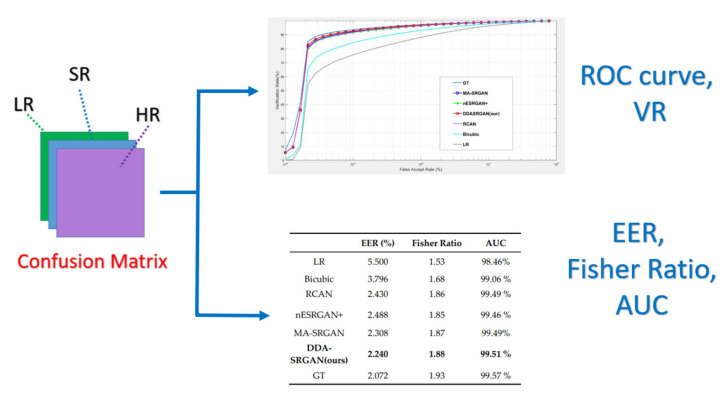
The flow chart of the performance evaluation stage.

The general procedure of biometric recognition can be mainly divided into two stages: the enrollment stage and matching stage. In this study, we further divide the detail of the proposed framework into four stages:

1. Enrollment stage: we load the HR image (iris image or face image) from the gallery set and take the image as input of recognition system to extract the corresponding features for enrolling the feature template of gallery set. Later on, the feature templates are stored in the local database. The enrollment stage is illustrated in Figure 6.
2. SR generation stage: we load the HR image from the probe set, and the corresponding LR images are obtained by downsampling the HR images with the scaling factor of ×4. We further feed the LR images into the SR generator to enhance the image resolution, as shown in the illustration in Figure 7.
3. Matching stage: the SR image obtained from the SR model will be further taken as the input of biometric recognition system, and the SR image feature will be extracted by the recognition system. After that, all of the feature templates extracted from the SR images will be compared by all the enrolled feature obtained from the local database to calculate the distances (Hamming distance or Euclidean distance). The resulting confusion matrix will be obtained by further calculating those distance. The matching stage is illustrated in Figure 8.
4. Performance evaluation stage: finally, the corresponding EER, Fisher ratio, and area under curve (AUC) information are calculated by the given confusion matrix, and the corresponding receiver operating characteristic (ROC) curves are plotted to visualize the overall performance of the recognition system as illustrated in Figure 9.

#### 4.4.2. Iris Recognition Procedure

In the experiment, the procedure of iris recognition is described into five stages: iris image acquisition, iris segmentation and normalization, feature extraction, and feature matching. In the first stage, HR iris images are acquired by NIR cameras, of which the wavelength can accurately reflect and capture the texture structure of the iris. The preprocessing including iris segmentation and iris coordinate transformation is executed so that the iris texture (which is the ROI for iris recognition purpose) in the original image will be transformed into the polar coordinate system, producing another representation of iris image in a rectangular shape. After that, the iris features will be extracted and converted into the vector of binary string, which is called the iris codes. The feature is compared with the already stored iris templates. In our experiments, the Harr-wavelet-based feature extraction method was used for feature extraction for iris images.

**Figure 10:**
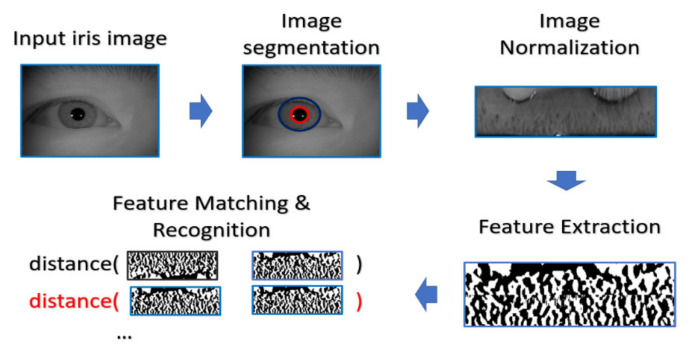
The classical procedure for iris recognition.

During the matching phase, the probe iris code is matched against all iris codes in the enrolled database. This matching is performed by computing the Hamming distance (HD) between the two iris codes. In our experiments, the threshold value of HD for acceptance or rejection is selected by considering the best EER. The overall process is depicted in Figure 10.

#### 4.4.3. Face Recognition Procedure

For face recognition, the face image is captured by the optical sensor, which can be either an RGB sensor or NIR sensor. The preprocessing including face detection and face alignment is executed so that the location of the face can be detected and the input face can be properly aligned. After that, depending on which model or classifier is chosen for the recognition, there exist different ways for feature extraction for face biometrics. In our experiment, we adopt a deep-learning-based model. We applied Dlib library [41] on the aligned face to extract the feature and encode it into a 128-dimensional vector and the encoded value will be normalized into the range [0, 1]. The feature extractor in Dlib applied a very deep residual network as the backbone to extract the face feature as well as encode the facial identities. In the matching phase, the probe face code is matched against all face templates in the enrolled database by computing the distance between two face codes.

**Figure 11:**
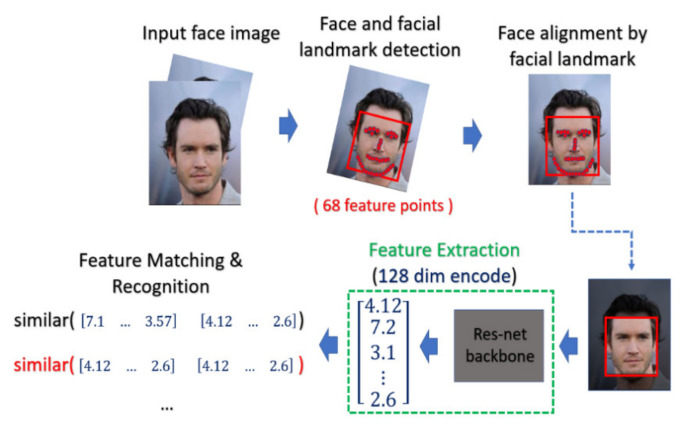
The classical procedure for face recognition.

In this paper, the threshold value of the distance is selected by considering the best EER. The overall process is depicted in Figure 11.

### 4.5. Details for Training and Parameters Tuning

At first, we also make use of the PSNR-oriented pre-trained model to initialize the generator. The loss weights for iris generation are 1.0 for perceptual loss, 0.03 for relativistic loss, and 0.05 for pixel-wise loss, respectively ($\lambda =1,\;\eta =3\times 10^{-2},\;\gamma =5\times 10^{-2}$) [31]. The learning rate was initially set to 0.0001 and trained for 10,000 epochs to achieve convergence. On the other hand, the loss weights for face generation are 1.3 for perceptual loss, 0.08 for relativistic loss, and 0.4 for pixel-wise loss ($\lambda =1.3,\;\eta =8\times 10^{-2},\;\gamma =4\times 10^{-1}$). The learning rate was initially set to 0.0001 and trained for 80,000 epochs to reach convergence.

In addition, the batch size is set to 4 for iris generation and 32 for face generation, due to the constraint of the GPU memory, and the general stability parameters of Adam optimizer fine-tune in training are $\beta_{1}=0.0009$ and $\beta_{2}=0.5$ including adding the AMS gradient to speed up the network convergence. The implementation is performed with Keras based on the TensorFlow backend and trained with 4 NVIDIA GeForce GTX1080 Ti GPUs.

## 5. Experimental Results

### 5.1. Downstream Task: Iris Recognition

**Figure 12:**
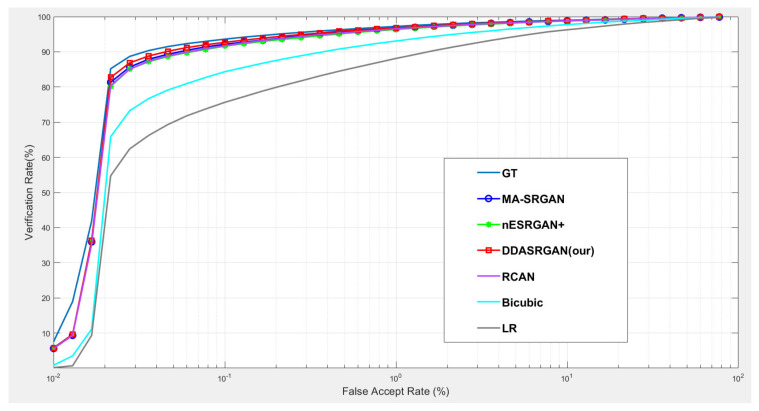
Comparison of ROC curves for each SR model on the CASIA-Iris dataset. The figure shows the various SR methods, and the deep-learning-based approaches show the outstanding performance compared to the traditional method (bicubic). Furthermore, the proposed DDA-SRGAN achieves the best performance and narrows the gap between the SR method and ground truth (GT).

**Figure 13:**
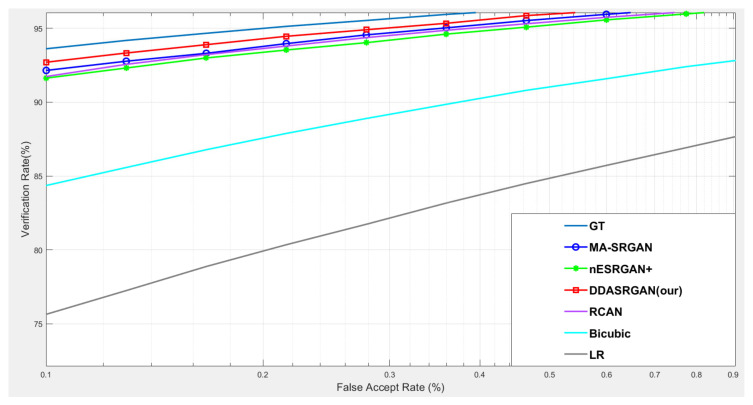
Detailed comparison of ROC curves for each SR model on the CASIA-Iris dataset. In the detailed comparison, we can find that the LR images downgrade the recognition accuracy almost 20% when FAR = 0.1%. The various SR approaches mitigate this gap, and the proposed method achieves the best performance among those approaches.

For iris recognition experiments, the ROC curve presents that the proposed DDA-SRGAN outperforms most of the SR methods, as shown in the illustration in Figure 12 and Figure 13, and the quantized value of each ROC curve also reflects on the AUC, of which a greater value indicates better performance. For the comparison between DDA-SRGAN and nESRGAN+, which is the latest SOTA in the SR field, our proposed method has better performance in terms of EER. Furthermore, our method compares with the MA-SRGAN, which is the latest proposed model in the field of biometric SR, also showing superiority with lower FAR, and achieves the lowest EER with 2.24%, as described in Table 4. To further understand the discriminating power between the authentic distribution and the imposter distribution, the fisher ratio is used to measure the different SR method, and the proposed DDA-SRGAN also shows the highest discriminating capability by presenting the largest distance between the distributions. Note that the ground truth (HR) images achieve an EER of 2.072%, and the gap between SR image and ground truth is very small.

### 5.2. Downstream Task: Face Recognition

**Figure 14:**
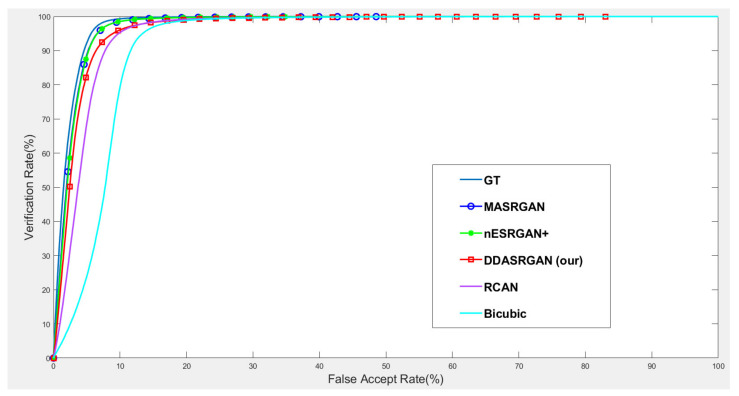
Comparison of ROC curves for each SR model on the CelebA dataset. The figure shows the various SR methods, and the deep-learning-based approaches show the outstanding performance compared to the traditional method (bicubic). The proposed DDA-SRGAN achieves an acceptable performance among all of the deep-learning approaches without the need for prior knowledge of ROI.

**Figure 15:**
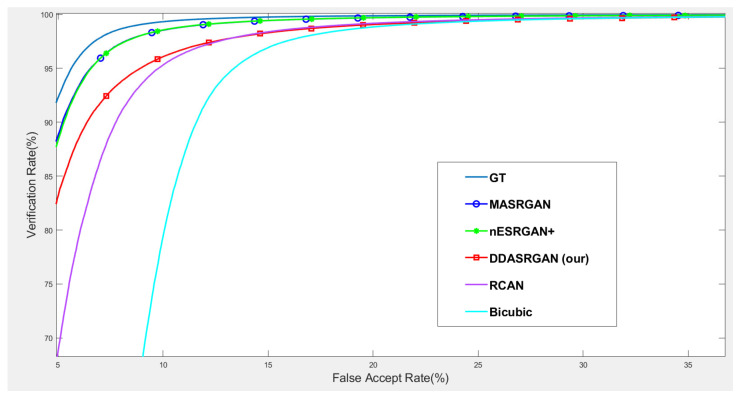
Detailed comparison of ROC curves for each SR model on the CelebA dataset. In the detailed comparison, we can find that the bicubic SR images downgrade the recognition accuracy to roughly 68% when FAR = 5%, and the various SR methods mitigate this gap. Among them, the proposed method achieves an acceptable performance without prior knowledge of ROI.

For face recognition experiments, the ROC curve presents that the proposed DDA-SRGAN outperforms some SR methods, as shown in the illustrations in Figure 14 and Figure 15. Although, DDA-SRGAN may not have better performance than nESRGAN+ as well as MA-SRGAN in the case of face recognition, the reason may be that multiple ROI regions of the face cause the model to barely optimize and converge to the global optimum. However, our proposed method still performs better than other kinds of attention-based method, such as RCAN, as it achieves lower EER and a higher Fisher ratio and AUC, as described in Table 5. Moreover, other SR methods, such as MA-SRGAN, require prior knowledge of the downstream tasks to indicate the ROI region.

### 5.3. Visual Evaluation

The objective metric of iris recognition has already presented the superiority of the proposed method in terms of the ROI feature, while the visual evaluation is still the mainstream method to judge the image quality in the SR field. Therefore, in this sub-section, we also present several visual comparisons in the iris images on the ROI part according to the domain knowledge.

**Figure 16:**
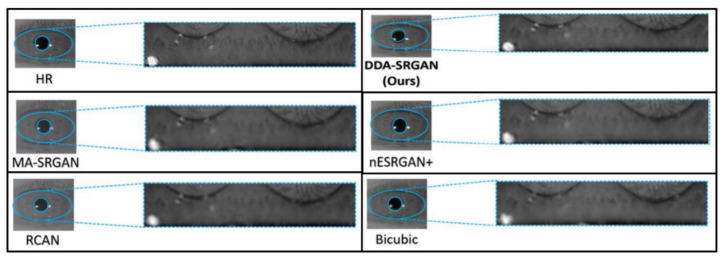
Visual comparison of the super-resolved image of “Rcls6_10” from CASIA dataset.

**Figure 17:**
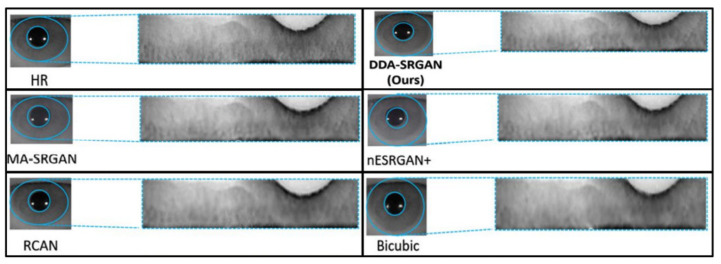
Visual comparison of the super-resolved image of “Rcls489_7” from CASIA dataset.

Figure 16 and Figure 17 demonstrate visual comparisons of normalized iris images. For both image sets, we observe that most of the compared methods cannot recover the iris texture and would suffer from the blurring artifacts. In contrast, our DDA-SRGAN can slightly alleviate such a side-effect and preserve the texture detail.

**Figure 18:**
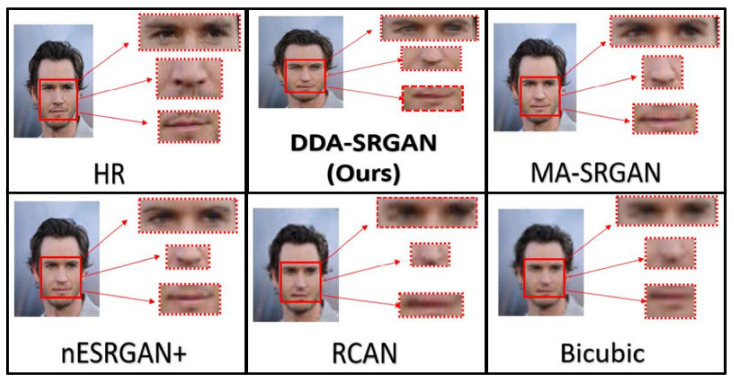
Visual comparison of the super-resolved image of “011006” from CelebA dataset.

**Figure 19:**
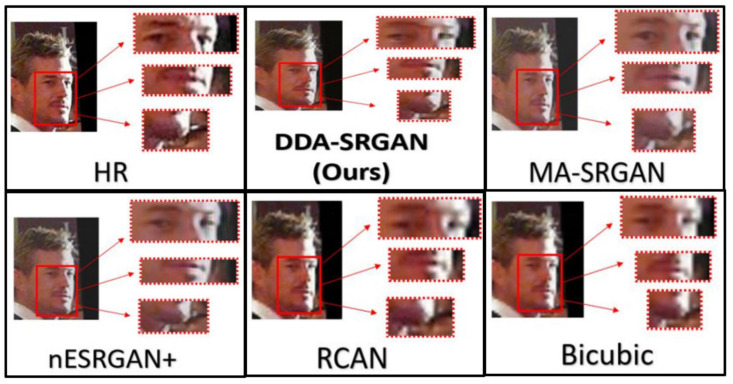
Visual comparison of the super-resolved image of “064049” from CelebA dataset.

Although the objective metric may not perform well in the case of face recognition, our proposed method still presents competitive results in the visual comparisons. In the experiment, we inspect the detail of the ROI feature by highlighting a few important regions on a face image, as illustrated in Figure 18 and Figure 19. For both image sets, we observe that some of the baseline methods produce the blurring artifacts nearby the region of facial landmarks, for example, RCAN produced blurred eyes, as shown in Figure 18. Some of the baseline methods, such as nESRGAN+ and MA-SRGAN, also generate images of which the color is less saturating (see Figure 19), while the proposed DDA-SRGAN produces more faithful results to describe the facial texture and preserve image color saturation.

### 5.4. Quantitative Evaluation

The quantitative metric is also used in the SR field to judge the image quality. Therefore, in this sub-section, we also present such results based on several commonly used metric such as PSNR and SSIM. To further measure the perceptual quality, we also compute the inception score to present the performance of image generation.

According to Table 6 and Table 7, we can observe that PSNR and SSIM almost present consistent results, which indicate that the bicubic and RCAN performs well when reconstructing the structural information of the images. On the other hand, the GAN-based methods produce worse results, whose distortion may be caused by super-resolving edges and textures which are not pleasing to the eyes. However, such reconstructed images may benefit the downstream task such as classification, recognition, etc., which is the novel perspective that we argue in this work. For the inception score, the perceptual score of the image is based on the VGG network. In Table 6, the details of the patch information are crucial for the classification; the proposed method achieves a competitive result and is only worse than ESRGAN+. In Table 7, the proposed method presents the worse case, which partially reveals a similar result of the face recognition in Table 5. The difference between the iris and face recognition is the number of potential ROI in the entire image. The iris recognition mainly focuses on the iris texture, which only exists in the single region nearby the pupil boundary. However, for face biometrics, there are multiple regions that influence the final recognition rate, such as noise, eyes, mouse, hair style, and each of them can be seem as one individual ROI in a face image. So, the proposed method is required to learn the multiple ROIs in the face SR hallucination task, which is harder to converge to the global optimum during the training. In other words, the weakness of the proposed method is that it may not be suitable for some special SR tasks when multiple ROIs exist.

### 5.5. The Comparison between the Previous Work

Some of the approaches in the SR field are based on the attention mechanism, whose network blocks may be quite similar with the proposed method. So, we attempt to briefly summarize the main difference between the proposed kernel module DDAB and the previous works. In the previous work, the RCAN as well as the RBAN are quite similar to the proposed method, which may cause the reader to be confused about the contribution of our work.

**Figure 20:**
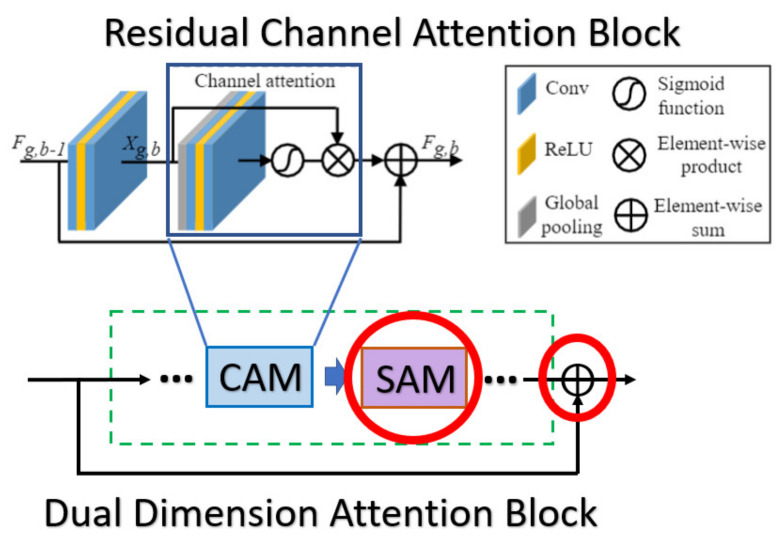
The main difference between the RCAB and the DDAB. The red solid circles indicate the additional parts in our module DDAB.

In the RCAN [9], the authors provide the residual channel attention block (RCAB), which makes use of the SE Block [31] to perform the channel attention with the additional residual connection as the kernel module to extract the feature in the LR images. While the proposed DDAB is composed of the CAM and SAM, we consider the feature correlation to not only be present across the channel but also in the content of each feature map. So, we combine the SAM followed by the CAM to further allow the DDAB to figure out the context correlation of each feature map. The main differences between the CAM as well as the DDAB are shown in Figure 20.

**Figure 21:**
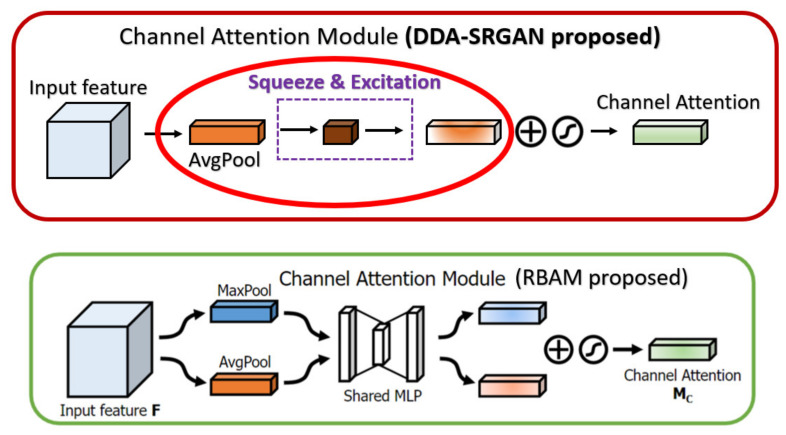
The main difference between the RBAM and DDAB in terms of channel attention module. The red solid circle indicates the different implementation in our module DDAB.

**Figure 22:**
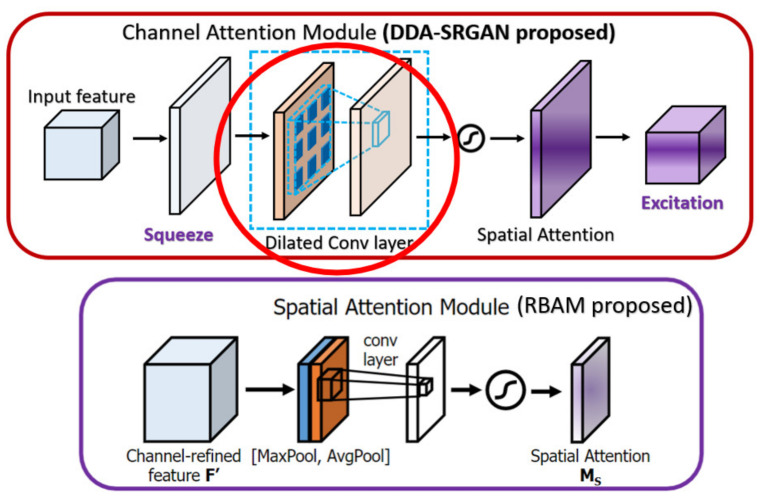
The main difference between the RBAM and DDAB in terms of spatial attention module. The red solid circle indicates the different implementation in our module DDAB.

In the CBAM [38], the channel attention module (also called CAM in CBAM) makes use of the max-pooling and the average-pooling to extract the cross-channel features and perform the concatenation, while we only use the global average pooling in our CAM to extract the global feature (cross-channel) without such post-processing. Since the max-pooling is prone to overfit in most cases, we eliminate such a mechanism to speed up the training procedure as well as to reduce the memory usage to store the intermediate features. Furthermore, the spatial attention module (also called SAM in CBAM) in the previous work only uses the convolution with a pooling layer to extract the contextual information (the value belongs to each feature map), but we further use the dilated convolution, which can enlarge the receptive field without losing too much information (unlike the method based on pooling) with a proper dilated rate, to extract the contextual information. The main difference between the CBAM as well as the DDAB are shown in Figure 21 and Figure 22, respectively.

## 6. Conclusions

We have proposed DDA-SRGAN for accurate super-resolution image reconstruction and demonstrated its effectiveness in the case of biometric recognition. Specifically, the dual-dimensional attention module automatically forces the generator to focus on the discriminative feature in the ROI region for enhancing the performance of biometric task. Furthermore, the proposed method allows the generators to reconstruct high-level semantic features, such as iris texture or facial details, which are crucial for enhancing the accuracy of the biometric system.

In the framework of IoT application, the security of the authentication system in BiometricIoT can be further enhanced by using the proposed SR method. Furthermore, the sensor node in the secured IoT network will be safe and the risk of the sensor nodes being compromised can be minimized. As a result, the proposed DDA-SRGAN outperforms the current SOTA (MA-SRGAN) in the task of iris by 0.5% VR in the large-scale biometric experiments and achieves competitive visual results in face recognition.

For the future works, we plan to combine the mask attention module into the proposed DDA-SRGAN to stabilize the performance of the face recognition task. It may also boost the performance of the iris recognition task by propagating the additional ROI information, resulting in a better SR method for mobile sensing networks.

## Figures and Tables

**Table 1:** Biometric dataset specification.

Dataset Type	CASIA (Iris Image)	CelebA (Face Image)
Number of Categories	2000	6000
Number of Images in each category	10	20
Image Resolution	640 × 480	160 × 200

**Table 2:** Dataset specifications of separation in the training phase.

Image Type	Categories	Number of Images per Class	Total Number of Images
Iris image	1000	10	10,000
Face Image	4200	20	84,000

**Table 3:** Data specifications of separation in the evaluation phase.

		Enrollment Dataset			Query Dataset	
Image Type	Categories	Number of Images per Class	Total Number of Images	Categories	Number of Images per Class	Total Number of Images
Iris image	1000	5	5000	1000	5	5000
Face Image	1800	10	18,000	1800	10	18,000

**Table 4:** The comparison with different SR method in iris recognition.

	EER (%)	Fisher Ratio	AUC	VR
LR	5.500	1.53	98.46%	75.62%
Bicubic	3.796	1.68	99.06%	84.35%
RCAN	2.430	1.86	99.49%	91.74%
nESRGAN+	2.488	1.85	99.46%	91.65%
MA-SRGAN	2.308	1.87	99.49%	92.23%
**DDA-SRGAN(ours)**	**2.240**	**1.88**	**99.51%**	**92.7%**
GT	2.072	1.93	99.57%	93.62%

**Table 5:** Comparison of the proposed method with different SR models for face recognition.

	EER (%)	Fisher Ratio	AUC	VR
Bicubic	11.310	1.16	92.34%	24.18%
RCAN	8.260	1.40	95.67%	68.74%
nESRGAN+	6.247	1.65	97.36%	87.71%
MA-SRGAN	6.237	1.64	97.41%	89.75
DDA-SRGAN (ours)	6.840	1.53	97.02%	84.24%
GT	5.506	1.86	97.91%	92.01%

**Table 6:** The comparison of the proposed method with different SR models for iris recognition.

	PSNR	SSIM (%)	Inception Score
Bicubic	33.91	89%	2.1
RCAN	34.88	89.5%	2.23
nESRGAN+	20.75	83.5%	2.35
MA-SRGAN	23.36	84.9%	2.29%
DDA-SRGAN (ours)	28.27	80%	2.3%
GT	inf	100%	-

**Table 7:** The comparison of the proposed method with different SR models for face recognition.

	PSNR	SSIM (%)	Inception Score
Bicubic	26.62	81.9%	3.18
RCAN	26.38	79.9%	3.11
nESRGAN+	22.4	76.6%	2.88
MA-SRGAN	21.22	74.5%	2.96
DDA-SRGAN (ours)	22.21	65.8%	2.73
GT	inf	100%	-

## Data Availability

In this study, we used a public iris dataset, CASIA-v4, which can be found here: http://www.cbsr.ia.ac.cn/china/Iris%20Databases%20CH.asp (accessed on 20 July 2021). We also used a public face dataset, CelebA, which can be found here: https://mmlab.ie.cuhk.edu.hk/projects/CelebA.html (accessed on 3 September 2021).

## References

[1] Number of Internet of Things (IoT) Connected Devices Worldwide from 2019 to 2030. https://www.statista.com/statistics/1183457/iot-connected-devices-worldwide/.

[2] Dhvani S., Vinayak H. IoT Based Biometrics Implementation on Raspberry Pi. Proceedings of the International Conference on Communication, Computing and Virtualization (ICCCV).

[3] Ehsan N.T., Adel N.T., Reza G., Rajkumar B. Integrated IoT and Cloud Environment for Fingerprint Recognition. Proceedings of the International Conference on Fog Computing and Internet of Things (ICFCIOT 2017).

[4] Farid F., Elkhodr M., Sabrina F., Ahamed F., Gide E. (2021). A Smart Biometric Identity Management Framework for Personalised IoT and Cloud Computing-Based Healthcare Services. Sensors.

[5] Kenneth L.A., Kah P.S. (2021). Biometrics-based Internet of Things and Big data design framework. Math. Biosci. Eng..

[6] Boucher A., Kyriakidis P.C., Cronkite-Ratcliff C. (2007). Geostatistical solutions for super-resolution land cover mapping. IEEE Trans. Geosci. Remote Sens..

[7] Yang W., Zhang X., Tian Y., Wang W., Xue J.-H., Liao Q. (2019). Deep learning for single image super-resolution: A brief review. IEEE Trans. Multimed..

[8] Goodfellow I., Pouget-Abadie J., Mirza M., Xu B., Warde-Farley D., Ozair S., Courville A., Bengio Y. (2014). Generative adversarial nets. Adv. Neural Inf. Process. Syst..

[9] Zhang Y., Li K., Li K., Wang L., Zhong B., Fu Y. Image super-resolution using very deep residual channel attention networks. Proceedings of the European Conference on Computer Vision (ECCV).

[10] Dai T., Zha H., Jiang Y., Xia S. Image super-resolution via residual block attention networks. Proceedings of the IEEE/CVF International Conference on Computer Vision (ICCV).

[11] Kim D., Kim M., Kwon G., Kim D. Progressive Face Super-Resolution via Attention to Facial Landmark. Proceedings of the 30th British Machine Vision Conference (BMVC).

[12] Li Q., Yu Z., Wang Y., Zheng H. (2020). TumorGAN: A Multi-Modal Data Augmentation Framework for Brain Tumor Segmentation. Sensors.

[13] Huang C.-E., Chang C.-C., Li Y.-H. (2021). Mask Attention-SRGAN for Mobile Sensing Networks. Sensors.

[14] Dong C., Loy C.C., He K., Tang X. Learning a deep convolutional network for image super-resolution. Proceedings of the European Conference on Computer Vision.

[15] Kim J., Lee J.K., Lee K.M. Accurate image super-resolution using very deep convolutional networks. Proceedings of the IEEE Conference on Computer Vision and Pattern Recognition 2016.

[16] Zhang Y., Tian Y., Kong Y., Zhong B., Fu Y. Residual dense network for image super-resolution. Proceedings of the IEEE Conference on Computer Vision and Pattern Recognition 2018.

[17] Lai W.-S., Huang J.-B., Ahuja N., Yang M.-H. Deep laplacian pyramid networks for fast and accurate super-resolution. Proceedings of the IEEE Conference on Computer Vision and Pattern Recognition 2017.

[18] Huang G., Liu Z., Van Der Maaten L., Weinberger K.Q. Densely connected convolutional networks. Proceedings of the IEEE Conference on Computer Vision and Pattern Recognition 2017.

[19] Kim J., Lee J.K., Lee K.M. Deeply-recursive convolutional network for image super-resolution. Proceedings of the IEEE Conference on Computer Vision and Pattern Recognition 2016.

[20] Tai Y., Yang J., Liu X. Image super-resolution via deep recursive residual network. Proceedings of the IEEE conference on Computer Vision and Pattern Recognition 2017.

[21] Haris M., Shakhnarovich G., Ukita N. Deep back-projection networks for super-resolution. Proceedings of the IEEE Conference on Computer Vision and Pattern Recognition 2018.

[22] Lim B., Son S., Kim H., Nah S., Mu Lee K. Enhanced deep residual networks for single image super-resolution. Proceedings of the IEEE Conference on Computer Vision and Pattern Recognition 2017.

[23] Tong T., Li G., Liu X., Gao Q. Image super-resolution using dense skip connections. Proceedings of the IEEE International Conference on Computer Vision 2017.

[24] Ledig C., Theis L., Huszár F., Caballero J., Cunningham A., Acosta A., Aitken A., Tejani A., Totz J., Wang Z. Photo-realistic single image super-resolution using a generative adversarial network. Proceedings of the IEEE Conference on Computer Vision and Pattern Recognition 2017.

[25] Johnson J., Alahi A., Fei-Fei L. Perceptual losses for real-time style transfer and super-resolution. Proceedings of the European Conference on Computer Vision.

[26] Bruna J., Sprechmann P., LeCu Y. (2015). Super-resolution with deep convolutional sufficient statistics. arXiv.

[27] Sajjadi M.S., Scholkopf B., Hirsch M. Enhancenet: Single image super-resolution through automated texture synthesis. Proceedings of the IEEE International Conference on Computer Vision 2017.

[28] Simonyan K., Zisserman A. Very deep convolutional networks for large-scale image recognition. Proceedings of the International Conference on Learning Representations.

[29] He K., Zhang X., Ren S., Sun J. Deep residual learning for image recognition. Proceedings of the IEEE Conference on Computer Vision and Pattern Recognition 2016.

[30] Rakotonirina N.C., Rasoanaivo A. Esrgan+: Further improving enhanced super-resolution generative adversarial network. Proceedings of the International Conference on Acoustics, Speech and Signal Processing.

[31] Hu J., Shen L., Sun G. Squeeze-and-excitation networks. Proceedings of the IEEE Conference on Computer Vision and Pattern Recognition 2018.

[32] Wang F., Jiang M., Qian C., Yang S., Li C., Zhang H., Wang X., Tang X. Residual attention network for image classification. Proceedings of the IEEE Conference on Computer Vision and Pattern Recognition 2017.

[33] Li K., Wu Z., Peng K.-C., Ernst J., Fu Y. Tell me where to look: Guided attention inference network. Proceedings of the IEEE Conference on Computer Vision and Pattern Recognition 2018.

[34] Cao C., Liu X., Yang Y., Yu Y., Wang J., Wang Z., Huang Y., Wang L., Huang C., Xu W. Look and think twice: Capturing top-down visual attention with feedback convolutional neural networks. Proceedings of the IEEE International Conference on Computer Vision 2015.

[35] Jaderberg M., Simonyan K., Zisserman A. (2015). Spatial transformer networks. Adv. Neural Inf. Process. Syst..

[36] Bluche T. (2016). Joint line segmentation and transcription for end-to-end handwritten paragraph recognition. Adv. Neural Inf. Process. Syst..

[37] Miech A., Laptev I., Sivic J. (2017). Learnable pooling with context gating for video classification. arXiv.

[38] Woo S., Park J., Lee J.Y., Kweon I.S. CBAM: Convolutional Block Attention Module. Proceedings of the European Conference on Computer Vision (ECCV) 2018.

[39] Institute of Automation, Chinese Academy of Science CASIA v4.0 Iris Image Database. http://www.cbsr.ia.ac.cn/china/Iris%20Databases%20CH.asp.

[40] Liu Z., Luo P., Wang X., Tang X. Deep Learning Face Attributes in the Wild. Proceedings of the International Conference on Computer Vision (ICCV) 2015.

[41] King D.E. (2009). Dlib-ml: A Machine Learning Toolkit. J. Mach. Learn. Res..

